# The role of water in the reversibility of thermal denaturation of lysozyme in solid and liquid states

**DOI:** 10.1016/j.bbrep.2021.101184

**Published:** 2021-12-07

**Authors:** Tuan Phan-Xuan, Ekaterina Bogdanova, Jens Sommertune, Anna Millqvist Fureby, Jonas Fransson, Ann E. Terry, Vitaly Kocherbitov

**Affiliations:** a Biomedical Science, Malmö University, Malmö, Sweden; b Biofilms Research Centrer for Biointerfaces, Sweden; c RISE Research Institutes of Sweden, Stockholm, Sweden; d SOBI Swedish Orphan Biovitrum, Stockholm, Sweden; e Max IV Laboratory, Lund University, Lund, Sweden

**Keywords:** Thermal denaturation/unfolding, Hydration, Solid state protein, Differential scanning calorimetry (DSC), Small and wide-angle X-ray scattering (SAXS/WAXS), Raman

## Abstract

Although unfolding of protein in the liquid state is relatively well studied, its mechanisms in the solid state, are much less understood. We evaluated the reversibility of thermal unfolding of lysozyme with respect to the water content using a combination of thermodynamic and structural techniques such as differential scanning calorimetry, synchrotron small and wide-angle X-ray scattering (SWAXS) and Raman spectroscopy. Analysis of the endothermic thermal transition obtained by DSC scans showed three distinct unfolding behaviors at different water contents. Using SWAXS and Raman spectroscopy, we investigated reversibility of the unfolding for each hydration regime for various structural levels including overall molecular shape, secondary structure, hydrophobic and hydrogen bonding interactions. In the substantially dehydrated state below 37 wt% of water the unfolding is an irreversible process and can be described by a kinetic approach; above 60 wt% the process is reversible, and the thermodynamic equilibrium approach is applied. In the intermediate range of water contents between 37 wt% and 60 wt%, the system is phase separated and the thermal denaturation involves two processes: melting of protein crystals and unfolding of protein molecules. A phase diagram of thermal unfolding/denaturation in lysozyme - water system was constructed based on the experimental data.

## Highlights

• Denaturation of lysozyme in solid and liquid is studied using SAXS, Raman and DSC.
• Denaturation of lysozyme in liquid is reversible, in solid state it is irreversible.
• A phase diagram of lysozyme-water system is constructed.

## 1 Introduction

Protein stability can be defined as the capability to maintain its structure in a folded well-defined conformation, or so-called native state, in an extended period [1]. Various factors such as heat, hydration and presence of denaturants can affect stability of the protein at various experimental or storage conditions. These factors are to different extents relevant for different applications. For example, protein stability with respect to hydration and dehydration is important for designing solid-state formulations of biologics [2]. Thermal stability of proteins is essential for the outcome of various biomedical and pharmaceutical applications such as thermal therapies and biopreservation [3]. Protein stability with respect to temperature and hydration are expected to be closely connected, however, the exact mechanisms of this relationship are yet to be fully understood.

Proteins in hydrated or dehydrated states possess different properties. When dehydrated, proteins lose their biochemical function due to changes in the protein structure and intrinsic dynamics. For instance, it was reported that the secondary structure of proteins such as the α-helix content decreases while the amount of β-sheet increases upon strong dehydration [[4], [5], [6]]. These and other structural transitions in proteins observed upon strong dehydration can be explained by the fact that the molecules adopt different conformations in order to reduce the air-protein interface and efficiently fill the space in the absence of water [7]. In our recent work [8], synchrotron small and wide-angle X-ray scattering (SWAXS) were used to characterize the structure of a model protein, lysozyme, in the solid state and its structural transition upon rehydration to the liquid state. In dried state, the scattering patterns showed a clear distinct feature of structural information which appears in a form of single broad peak at q = 2.5 nm^-1^, which indicates that the molecules in the amorphous state must undergo deformations to be able to closely pack together. Above the hydration threshold of 35 wt%, the native structure of the protein is recovered. These changes in protein morphology may play a crucial role in the thermal stability of a protein. In particular they can shift the denaturation conditions compared to the native state or facilitate non-equilibrium effects in the denaturation processes.

The most dramatic change during heating is denaturation - a transition characterized by changes in the tertiary structure and the loss of functional activity. A closely related but not equivalent term is unfolding, which emphasizes the structural changes in the protein. Contrary to what might be expected from this term, thermal unfolding of proteins (for example observed in differential scanning calorimetry experiments) does not lead to a coil-like structure. Instead, so-called molted globule state characterized by substantial structural order is formed [9].

Thermal denaturation or unfolding of globular proteins can be described by thermodynamic approach. Small globular proteins in dilute conditions usually show unfolding behavior in which the folded and unfolded states are the only two states that are the most populated during the transition. It also means that the unfolding process is reversible under the condition that their chemical structures are not damaged, i.e., no covalent bonds break or form. While the literature on thermal unfolding of globular proteins (and lysozyme as a model protein in particular) in liquid is broad, (see for instance some classical works by Privalov and co-workers [[10], [11], [12], [13]]), the mechanisms of protein denaturation in the solid state are largely unexplored.

In this work, we will present a thermodynamic and structural investigation of the effect of hydration on thermal denaturation of lysozyme to shed some light on the relationship between protein structure and moisture in relation to the protein's thermal stability.

## 2 Materials and methods

### 2.1 Materials

Lysozyme from chicken egg white (CAS number 12650-88-3, product number L6876, Lot SLBL 7146V) was purchased from Sigma-Aldrich and used as received. Milli-Q purified water was used for all experiments.

Preparation of rehydrated lysozyme:

Lysozyme was first dried in a vacuum pistol for 48 h using molecular sieves (type 3Å) as a sorbent. The amount of residual water in the powder after drying was determined using Thermalgravimetric Analysis (Q500, TA Instrument). A small amount (5–10 mg) of the dry lysozyme powder was rehydrated to the desired water content to produce samples covering the hydration range from 2.5 wt% to 99.6 wt% water content. Specifically, the re-hydration of lysozyme was carried out depending on the measurements and will be described in detail in the next section.

### 2.2 Differential scanning calorimetry

Differential scanning calorimetry measurements were performed with DSC 1 (Mettler Toledo, Switzerland) to study the calorimetric events associated with the denaturation of lysozyme. Temperature calibration and heat flow calibration were done using indium. Calorimetric enthalpies were determined in STARe Software by ISO standard (ISO 11357–2:1999). An empty aluminum crucible was used as a reference.

In case of dry samples, they were placed in 40 μm aluminum pans, hermetically sealed in nitrogen atmosphere with relative humidity less than 5% RH. We used equilibration or interrupted equilibration of dried material at various controlled relative humidities using saturated salt solutions LiCl (a_w_ = 0.11), MgCl_2_ (a_w_ = 0.33), Mg(NO_3_)_2_ (a_w_ = 0.53), NaCl (a_w_ = 0.75), KCl (a_w_ = 0.84), K_2_SO_4_ (a_w_ = 0.97) [14] to obtain different water contents in solid samples. The samples with water contents more than 30 wt% were prepared in the aluminum pans by weighing the lysozyme powder, adding the required amounts of liquid MQ water (ELGA, Purelab Flex) and sealing the pan.

DSC measurements were performed in a scanning mode. In a typical experiment, the sample was heated up to 100 °C for liquid state or 140 °C for solid state samples at a scanning rate of 1 °C/min. The DSC data was exported into MATLAB (MathWorks, Inc) to further data processing. It was first corrected by subtracting a baseline which is determined by fitting a line through individual selected points where no thermal events is observed.

### 2.3 Raman spectroscopy measurements

Raman spectroscopy was performed on a Witec Alpha 300RAS (Witec GmbH, Ulm, Germany), using a 532 nm laser for excitation. The samples were placed in a cavity microscope slide, covered with a cover slip and sealed with nail polish in order to keep the water content as stable as possible during the measurements. The temperature was controlled using a Linkam PE 94 Peltier stage (Linkam Scientific Instruments, Tadworth, England). The samples were heated/cooled at 1 °C/min and kept constant during the measurements at the specified temperatures. The sample mass was checked after the heat treatment and showed no water loss.

Raman spectra were recorded with 1800 g/mm grating between 650 and 1700 cm^-1^. The Raman spectra were collected at three different positions for each sample and temperature, each spectrum was collected for 100 s in total. The resulting three spectra were averaged, corrected for cosmic rays and background in the Witec Project plus 5.1 software.

### 2.4 Simultaneous SAXS/WAXS measurements

SWAXS measurements were carried out at the NCD-SWEET beamline at ALBA synchrotron (Spain) using an X-ray wavelength of 1 Å. Two-dimensional SAXS and WAXS images were recorded in a SAXS detector PILATUS 1 M (Dectris) located at a sample-detector distance of 2.69 m and a WAXS detector LX255-HS (Rayonix) at 0.12 m sample-detector distance, respectively. The scattering vector, q, was calibrated with a silver behenate sample. Reported scattering profiles I(q) were obtained by radially averaging of 2D SAXS images using the data reduction scripts at the beamline. With this set-up, a broad range of q-values: q = 0.007–5.6 nm-1 for SAXS and q = 5–85 nm-1 for WAXS was covered.

The Linkam stage (HFSX 350, Linkam, UK) was used to control the heating/cooling procedure. Samples were heated from 25 °C to above the unfolding temperature of lysozyme as determined by DSC. The heating rate was set to 2 °C/min. After heating, the samples were immediately cooled to 25 °C with 30 °C/min cooling rate.

## 3 Results and discussion

### 3.1 Calorimetric measurements of the protein thermal denaturation

#### 3.1.1 Dependence of the denaturation temperature and enthalpy on the hydration level

**Fig. 1:**
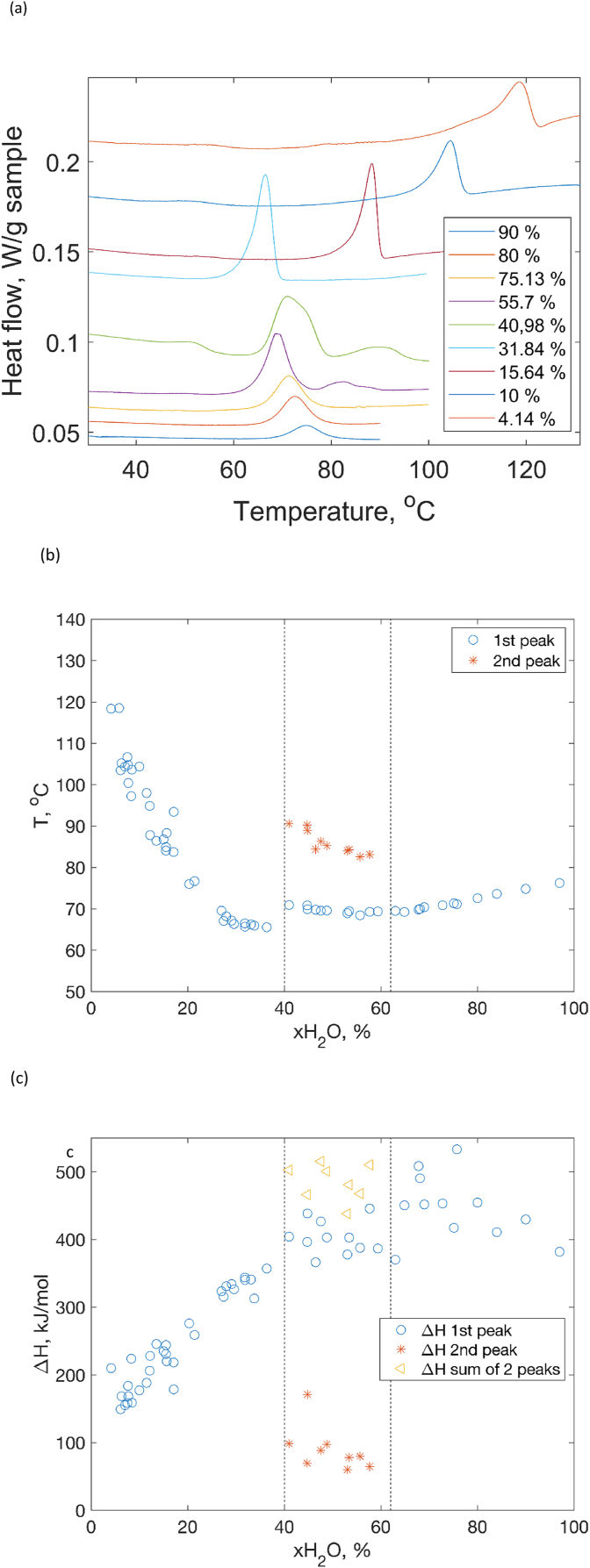
(a) Heat flow curves of DSC during a thermal denaturation of lysozyme at different water contents, (b) Temperature at the peak positions in the DSC curves as a function of the water content, (c) Integrated calorimetric enthalpy per mol of protein as a function of the water content.

The heat-induced protein denaturation can be observed experimentally by studying the thermodynamic properties of the system. During a temperature increase, different intramolecular interactions that keep the protein in its native functional states such as hydrogen bonds, hydrophobic, van der Waals and ionic interactions gradually weaken and can eventually be broken. This leads to unfolding and possibly aggregation of the proteins. In this process, energy is absorbed by the system thus results in endothermic events in the DSC data. In Fig. 1a, typical DSC scan patterns obtained for lysozyme are shown and could be divided into three different regimes depending on the water content:

• Below 37 wt%: the samples are in a solid amorphous powder state, one melting event with an asymmetric shape is observed
• Between 37 wt% - 60 wt%: the samples appear in a soft paste state of a two-phase system consisting of a solution and crystals of lysozyme. In this intermediate range, another additional melting peak at higher temperatures is observed which is associated with the melting of the lysozyme crystals
• Above 60 wt%: The samples are in a clear liquid aqueous solution state. The DSC curves show only one peak with a symmetric shape

In Fig. 1b the denaturation temperature obtained from the peak of the melting events is plotted as a function of the water content. The results could be divided into three different regimes depending on the water content: T_den_ decreases drastically with increasing water content below 37 wt%. It then remains nearly constant at water contents of 40 wt% – 57 wt% for the 1st denaturation peak temperature. The 2nd peak which is associated with the melting of crystals appears at a higher temperature and decreases with increasing water content. Finally, in the liquid state at a water content above 57 wt%, T_den_ slightly increases with increasing the water content.

The enthalpy of the denaturation can be determined experimentally by integrating the area below the denaturation peak on the heat capacity curve. This value is referred to as the calorimetric enthalpy (ΔH_cal_). In Fig. 1c, the hydration dependence of the calorimetric enthalpy of the denaturation process in J/mol of protein are shown. Three different regimes of the evolution of the enthalpy could be observed which is also consistent with the change in denaturation temperature. In the amorphous regime below 40 wt% of water the enthalpy value increases with increasing the water content. It then reaches a stable value, whereas a small contribution from the 2nd peak shows a slightly decreasing trend. In the liquid state regime, although the datapoints are scattered, a somewhat decreasing trend can be seen.

In the following sections we will analyze the complex calorimetric behavior observed in these experiments from point of view reversibility of unfolding and denaturation processes.

#### 3.1.2 Equilibrium unfolding in liquid phase system

In dilute solution, the denaturation of small proteins such as lysozyme is usually described by a two-state model, which assumes that only two protein conformations (native and denatured) coexist in equilibrium:

(1) $$N\overset{K}{↔}D$$

where *N* and *D* are the native and denatured states of protein, respectively.

When taking into account the role of water in this reversible process, Eq. (1) can be rewritten as follows:

$$nH_{2}O+N\overset{K}{↔}D$$

The equilibrium constant, *K*, for a two-state model can be written:

(2) $$K\left(T\right)=\frac{\left[D\right]}{\left[N\right]\cdot a_{w}^{n}}.$$

For a dilute system when $a_{w}^{n}\approx 1$ , or when the activity of water does not change significantly in the unfolding process, $a_{w}^{n}$ can be included in the value of the equilibrium constant. Then the equilibrium constant is determined using the degree of unfolding α: $K\left(T\right)=\frac{\;\alpha }{1-\alpha }$. Using the well-known relation between the Gibbs energy and the enthalpy ΔG=ΔH−TΔS, one could obtain the following equation:

(3) $$\Delta H_{vH}\left(\frac{1}{T}-\frac{1}{T^{0}}\right)=-RT\text{ln}K\left(T\right)$$

**Fig. 2:**
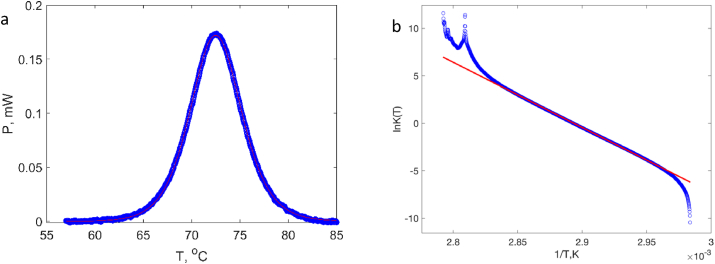
Calorimetric data of the unfolding of lysozyme xH_2_O = 80 wt% analyzed using van ’t Hoff model. (a) The experimental DSC data (blue circles) and their non-linear fitting using van ’t Hoff equation (red curve). (b) The same DSC data plotted in coordinates of Equation (3). (For interpretation of the references to colour in this figure legend, the reader is referred to the Web version of this article.)

Or in a differential representation:

(4) $$\frac{dlnK\left(T\right)}{d\left(\frac{1}{T}\right)}=-\frac{\Delta H_{vH}}{R}$$

which is often referred to as the van't Hoff equation where $\Delta H_{vH}$ is the van't Hoff enthalpy. The van't Hoff enthalpy can be calculated by plotting the logarithm of $\frac{\;\alpha }{1-\alpha }$ versus reciprocal temperature or alternatively by non-linear fitting if the DSC data on heat capacity or thermal power, see Fig. 2, where an example for 80 wt% of water is shown.

All the data in the liquid state regime are fitted perfectly by the van't Hoff model. It is therefore confirmed that the denaturation process is reversible in the liquid state. This is in a good agreement with other thermodynamic studies on lysozyme and other single-domain small compact globular proteins [12,15].

**Fig. 3:**
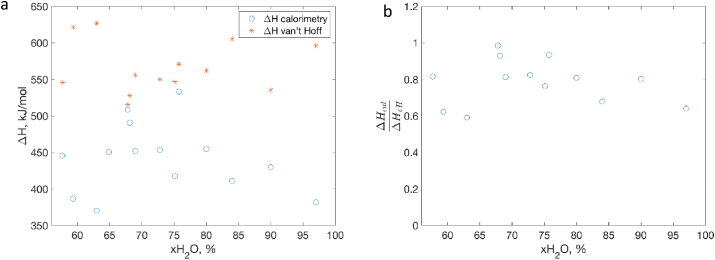
(a) Evolution of the unfolding enthalpy as determined by DSC and by fitting using van't Hoff model for lysozyme in the liquid state above 57% of water content. (b) The ratio of the calorimetric enthalpy and van't Hoff enthalpy in the same hydration regime.

When the calorimetric data are plotted in a van't Hoff representation as lnK(T) vs $\;\frac{1}{T}$ , they can be described by a single straight line (see an example in Fig. 2b) and from the slope, we can deduce the van't Hoff unfolding enthalpy (Fig. 3a). The values of enthalpy obtained from the fit are in the same range as the values that reported in literature. However, our calorimetric enthalpies are typically lower than the ones determined using van't Hoff model as well as experimental data in literature. This results in a ratio of $\frac{\Delta H_{cal}}{\Delta H_{vH}}$ < 1 (Fig. 3b). Theoretically, in a two-state denaturation process, the van't Hoff enthalpy should be equal to the calorimetric enthalpy, i.e. $\frac{\Delta H_{cal}}{\Delta H_{vH}}$ = 1 [11]. This ratio is however found higher than unity in many studies on the thermal denaturation of lysozyme (see Table 1). The discrepancy in the calorimetric enthalpy compared to literature could be due to technical reasons. In particular, accurate measurements of calorimetric unfolding enthalpies should be performed using a specialized liquid-state DSCs designed for studies in dilute solutions. Furthermore, parameters of the liquid state such as pH strongly influence the results (see some examples in Table 1), and should be accurately controlled, which was impossible to do in the present work. The aim of this work was to compare unfolding/denaturation processes in the whole range of water contents from dry solid sate to liquid solution, which can only be done using a solid-state DSC without adding a buffer. Despite certain discrepancy in calorimetric enthalpy values, the DSC peak shapes and the van't Hoff enthalpy data clearly show that the process of thermal denaturation of lysozyme is reversible not only in the dilute solution, but also in concentrated liquid samples.

**Table 1:** Comparison of the literature values of calorimetric and van't Hoff enthalpies.

Sample	pH	T_m_ (^o^C)	$\Delta H_{\mathrm{cal}}$ (kJ/mol)	$\Delta H_{\mathrm{vH}}$ (kJ/mol)	$\frac{\Delta H_{\mathrm{cal}}}{\Delta H_{\mathrm{vH}}}$	Ref
**Dilute solution**	3.3	65–75	502.080	305.432	1.64	[16,17]
**xH**_**2**_**O = 83 wt%**	6.24	74.1	635.131	535.552	1.19	[15]
**Dilute solution**	5	75.9	687.850	514.632	1.34	[18]
**Dilute solution**	2.5	63.7	674.265	473.629	1.37	[10]
**Dilute solution**	4.5	78.8	705.422	613.374	1.15	[11]
**Dilute solution**	–	–	–	–	1.05	[12]

#### 3.1.3 Kinetics of unfolding in solid phase

**Fig. 4:**
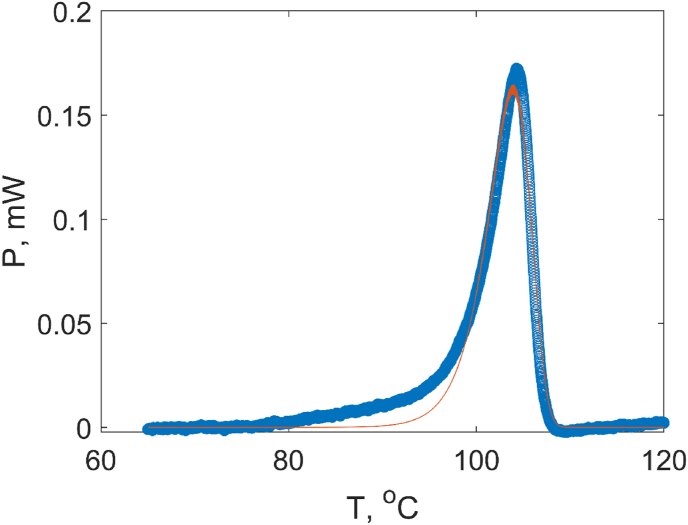
Calorimetric data of the protein unfolding analyzed using the kinetic approach. The experimental DSC data for lysozyme xH_2_O = 17 wt% (blue circles) and their fitting using Arrhenius model using equation (8) (red curve). (For interpretation of the references to colour in this figure legend, the reader is referred to the Web version of this article.)

The DSC data obtained on solid state lysozyme samples exhibit denaturation peaks with clearly unsymmetric shapes (Fig. 4). These peaks cannot be described with equations for reversible equilibrium transitions discussed above, which suggests that the denaturation in the solid state is irreversible. Irreversible processes should be described using a kinetic rather than thermodynamic approach. The kinetic approach is useful for describing many biotechnological applications of proteins (where kinetic stability is essential) as well as to develop understanding of protein misfolding diseases [19,20]. Most of the studies on kinetics approach were reported for various protein systems at high water contents (see reviewed by Ref. [21]). For transitions of biopolymers is a solid state, applicability of thermodynamic and kinetic approaches was discussed based on the DSC data for gelatinization processes of starch [22]. In the kinetic approach, the denaturation is assumed to be an irreversible reaction as follows:

(5) $$mH_{2}O+N\overset{k}{\to }D$$

where *m* is the number of water molecules involved in the reaction.

The rate of reaction v is defined through the degree of conversion:

(6) $$v=\;\frac{d\alpha }{dt}=k\left(T\right)\cdot \left(1-\alpha \right)\cdot a_{w}^{m}$$

where $a_{w}$ is the water activity; *k* is the reaction rate constant, *t* is time. For the case of constant water activity, the reaction is considered as of being a pseudo first order reaction.

(7) $$v=\;\frac{d\alpha }{dt}=k_{1}\left(T,a_{w}\right)\cdot \left(1-\alpha \right)$$

where $k_{1}\left(T,a_{w}\right)=k\left(T\right)\cdot a_{w}^{m}$ is the temperature and water activity dependent reaction rate constant. The temperature dependence of the reaction rate constant can be described by the Arrhenius representation:

(8) $$k_{1}\left(T,\;a_{w}\right)=A\left(a_{w}\right)e^{\frac{Ea}{RT}}$$

where *Ea* is the activation energy, $A\left(a_{w}\right)$ is a pre-exponential factor.

In the solid state, the value of $a_{w}$ is substantially smaller than 1 [7,23], thus the value of $k_{1}\left(T\right)$ decreases upon decrease of water activity. The number of water molecules *m* that is required to unfold lysozyme molecules can be very high, which further strengthens the dependence of $k_{1}$ on water activity. Thus, to provide the values of reaction constant that would result in significant reaction rates, the denaturation temperature should increase (eq (8)). This effect we clearly seen in Fig. 1 b, where a sharp rise of denaturation temperature is seen upon dehydration in the left-hand side of the plot.

By combining equations (7), (8), one can determine the dependence of the degree of conversion on temperature in an irreversible unfolding process. There is an extensive literature on how to calculate different parameters in equation (8). In this study, we have evaluated the Borchard-Daniels method [24] in which the combination of equations (7), (8) is written in a logarithmic representation:

(9a) $$\text{ln}\frac{d\alpha }{dt\left(1-\alpha \right)}=lnA-\frac{Ea}{RT}$$

The activation energy can then be determined by calculating the slope of the dependence of $\text{ln}\frac{d\alpha }{dt\left(1-\alpha \right)}$ , which can be considered as the logarithm of the reaction rate constant $k_{1}\left(T,a_{w}\right)$.

**Fig. 5:**
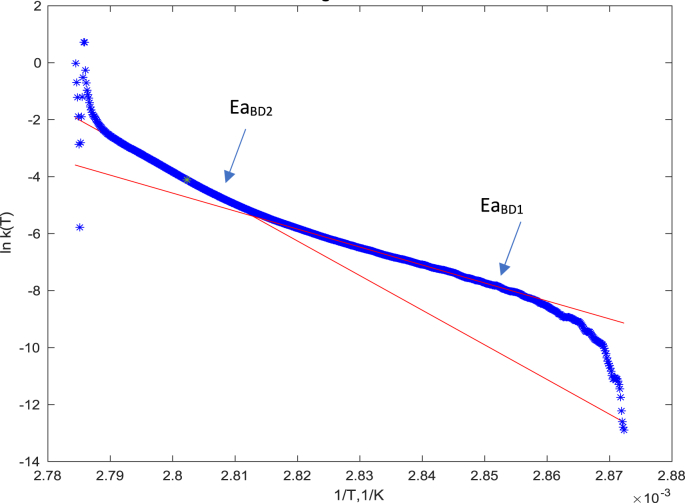
An example of Borchardt - Daniels plot of the reciprocal temperature dependence of the log of reaction rate constant for lysozyme xH_2_O = 17%.wt. The solid lines are the fits using the equation (9a), (9b) for two different temperature ranges.

Another way to describe the data and obtain the parameters of the kinetic model (in particular activation energy) is to use a direct fitting of the DSC heat capacity or thermal power data using the Arrhenius model expressed by eqs (7), (8)). Fig. 4 shows an example of the fitting results using this approach. Although the model reproduces the general shape of the peak, the data are not perfectly fitted in the lower temperature part of the transition. To understand the reason for this discrepancy and to get further insights into the mechanisms of denaturation processes we used the Borchard-Daniels method (eq. (9a), (9b)) for presentation of the same data, see Fig. 5. In these coordinates (the logarithm of kinetic constant as a function of reciprocal temperature), two regimes corresponding to two different temperature ranges could be identified. From the slopes of the linear parts of the curves, one could calculate the activation energies for both temperature ranges.

**Fig. 6:**
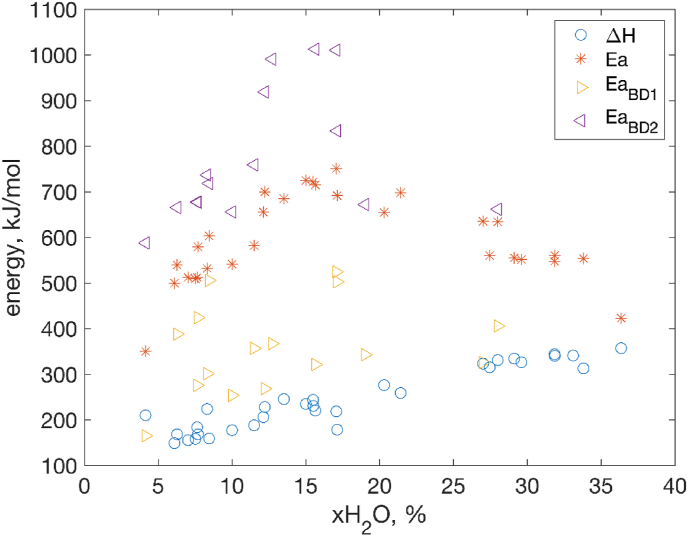
Comparison of energies and enthalpies of unfolding/denaturation processes obtained by different methods. Calorimetric enthalpy (blue circles) and activation energy obtained from Arrhenius model fitting (red stars) and from Borchardt Daniels method (yellow and purple triangles). (For interpretation of the references to colour in this figure legend, the reader is referred to the Web version of this article.)

The values of activation energies determined by peak fitting and Borchardt Daniels models together with calorimetric enthalpies are plotted as a function of the water contents in Fig. 6. The activation energy (Ea_BD2_) in the 2nd temperature regime is found to be the highest while it is lower in the 1st temperature regime (Ea_BD1_). The existence of two activation energies suggests that there might be more than one protein population in the system that are unfolded differently. This hypothesis might be plausible as it is supported by the fact that two different structures (packing arrangements) of protein are found in the amorphous solid at ambient temperature [8] that might uptake water differently.

Another alternative does not assume a difference in the states of protein molecules in the native state. Instead, a fraction of protein molecules takes up an additional amount of water during denaturation in the first temperature regime thus dehydrating the system. The remaining fraction of protein molecules has access to a lower amount of water, which results in an unfolding at higher temperature with higher activation energy.

The values of the activation energy determined by the peak fitting model lay in between the two values by Borchardt Daniels model. The lowest value of energy is the experimental calorimetric enthalpy. The difference in the values of the activation energy *Ea* and calorimetric enthalpy Δ*H* in the amorphous solid state is due to the fact that they represent different aspects of an unfolding reaction: *Ea* is the energy required to start the unfolding reaction and reach an intermediate state with a higher energy while Δ*H* reflects the final energy balance in system after the reaction is complete.

There is another aspect that can further increase the difference between the activation energy and the calorimetric enthalpy. In calculations based on eqs (7), (8), (9a), (9b)) the molecular mass of the protein is not explicitly used. Indeed, the degree of conversion at certain temperature is calculated from the relation of the peak area to the left from this temperature point to the whole peak area. Therefore, the activation energy is not necessarily related to a transition in a single protein molecule. If the protein molecules interact strongly (which is the case in the solid state), the observed activation energy can be related to a transition in a cooperative domain consisted of several molecules. For calculations of van't Hoff enthalpy (eqs (2), (3), (4))) the protein molecular mass is not explicitly used either. These calculations are, however, performed on a dilute solution data, where the molecules are well-separated by water hence the cooperative domain is one protein molecule.

#### 3.1.4 The two-phase system

**Fig. 7:**
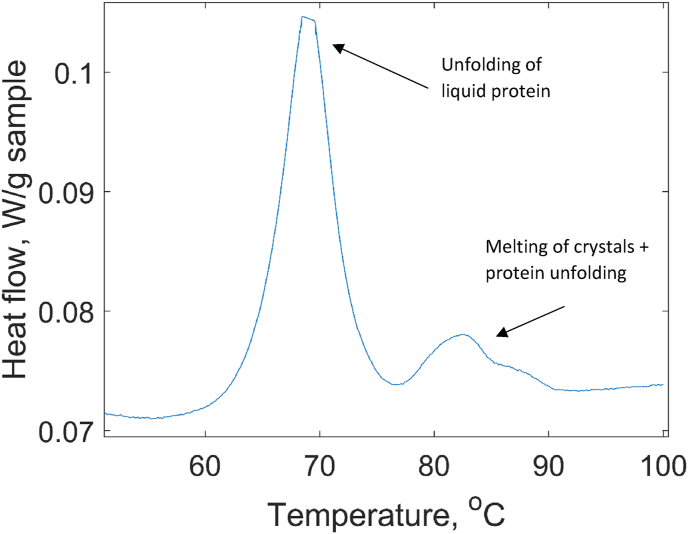
Heat flow curves of DSC during a thermal denaturation of lysozyme for x H_2_O = 55.7 wt % in which a phase separation into liquid and crystalline is observed.

At water contents between 40 wt% and 60 wt%, two endothermic peaks were seen in the DSC data (Fig. 1, Fig. 7). As reported in our previous work [8], the protein samples in this concentration regime undergo a phase separation. By examining X-ray scattering patterns of each phase, we concluded that the top phase contains liquid solution of native lysozyme while the bottom phase is the crystalline protein (see the section 3.3 below). Thus, the transformations in the sample in this concentration range include not only thermal denaturation of protein molecules, but also melting or dissolution of lysozyme crystals. From the Fig. 1 b it is clear that the first peak corresponds to the same process that occurs at water contents above 60 wt%, i.e. unfolding of lysozyme in liquid solution. The second peak represents a combination of two processes: crystals melting and protein unfolding. A deeper understanding of the interplay between the phase transformation and unfolding can be obtained using a phase diagram approach, which will be presented in the next section.

### 3.2 The phase diagram of lysozyme – water system

**Fig. 8:**
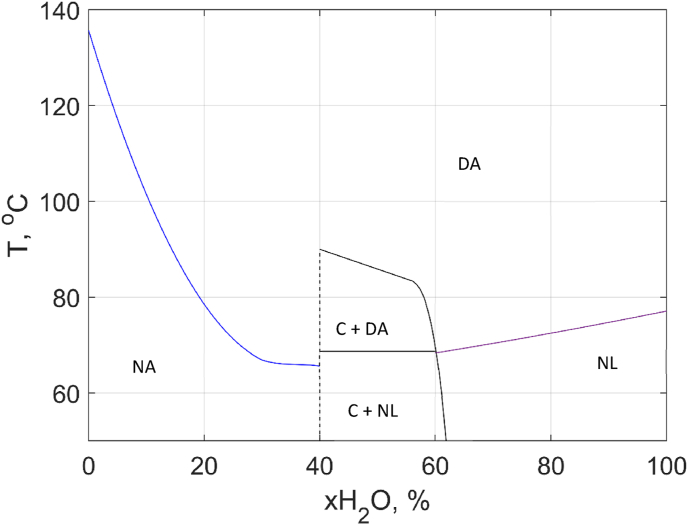
Thermal unfolding phase diagram of the lysozyme – water system. The phase boundaries were determined based on the DSC data. Labels in the phase diagram represent different states of the protein as a function of temperature and water content. NA: native amorphous, DA: denatured amorphous, C: crystals, DA: denatured amorphous, NL: native liquid.

The DSC data were used to construct a thermal unfolding phase diagram for the lysozyme – water system, see Fig. 8. On the right-hand side of the phase diagram, where one liquid phase is present, lysozyme molecules are in the native state below the unfolding temperature and in the unfolded state at above the temperature line. In the middle of the phase diagram, a two-phase region where crystalline lysozyme coexists with liquid solution of native lysozyme. As the temperature increases above the 1st transition line, the proteins in the liquid phase are transformed into the denatured state. With further increase of temperature, the crystals are melted and at the same time converted into the denatured state (Fig. 7). As the temperature goes above the 2nd transition line, the melting of crystals is completed, and the system consists of liquid solution of denatured protein. Finally, on the left-hand side, lysozyme molecules are in an amorphous state (that can be considered as native for the dehydrated conditions, denoted as NA in the phase diagram). With increasing the temperature to above the unfolding temperature, the native proteins are transformed into the denatured state. The denatured state of the protein we denoted ad DA (denatured amorphous), which can include both liquid and solid amorphous states and their combinations depending on water contents and conditions of the experiment.

In the liquid state regime in which the thermal unfolding of lysozyme is reversible, in order to understand the lysozyme -water system phase diagram, it is necessary to refer to the phase rule. According to the phase rule, the number of degrees of freedom of the system (the number of intensive thermodynamic state parameters sufficient to define equilibrium) is calculated as follows:

(9b) f=n−r+2

where *f* is the number of degrees of freedom, *n* is the number of independent components, *r* is the number of phases, 2 corresponds to temperature and pressure.

In our case, as *P* = const and *n* = 2 (as the system consists of water and protein):

(10) f=3−r

Strictly speaking, there are three different types of molecules in the system: water, native and denatured protein. On the other hand, the two types of protein molecules are in equilibrium in the liquid solution, which makes their chemical potentials dependent on each other:

$$\mu_{N}^{liq}=\mu_{D}^{liq}$$

Hence the number of independent components in the aqueous solution is two (water and protein).

In the liquid state below or above the transition line one phase is present in the system (solution of either native or denatured protein), the degree of freedom is 2, meaning that there are 2 independent parameters that define the thermodynamic properties of the systems, such as temperature and water content.

At the equilibrium condition, i.e., along the transition line from liquid native to denatured, strictly speaking, the number of phases is still one and the denaturation line does not represent a phase transition. However, an additional condition is that the transition point obtained from DSC data is the point where the concentrations of the native and denatured protein molecules are equal:

(11) $$C_{N}^{L}=C_{D}^{L}$$

In which $C_{N}^{L}$, $C_{D}^{L}$ and the concentration and denatured protein in the liquid phase, respectively. Thus, with the additional condition of equal concentrations, the degree of freedom is equal to one. It means that for a given water content the system thermodynamically defines the equilibrium transition temperature.

In the two-phase regime below the first transition line the system consists of a crystalline phase and a liquid solution of native lysozyme, the system has f=3−2= 1° of freedom, corresponding to a line in the phase diagram. According to the lever rule, at the lower limit xH_2_O = 40 wt%, the system consists of only crystals while at the higher end, it is only a liquid. If the water content shifts to lower value, the equilibrium will also shift to a higher fraction of crystals in the system. We should, however, note that the vertical line at 40 wt% in the phase diagram might not necessarily correspond to the water content in the crystals. Strictly speaking, it shows the threshold of the water content needed for formation of crystals, which due to kinetic reasons can be different from the water content in the crystalline phase.

The 1st transition line above the two-phase regime corresponds to two phases (crystals and liquid with equal concentrations of native and denatured protein molecules). This results in 0° of freedom meaning that the temperature is fixed in the whole range of the coexistence of crystals with the liquid where the degree of conversion equal to 0.5.

The area between the 1st and 2nd transition line in the two-phase regime consists of a mixture of native crystalline and denatured liquid lysozyme, thus there is 1° of freedom in the system as similar to the area below the 1st transition line. As the temperature gradually increases, more crystalline lysozyme is converted to the denatured protein in the liquid state.

The slope of the phase boundary *dT/dx* in the phase diagram is directly related to the DSC signal for a two-phase system [25]:

(12) $$C_{p}\left(1+2\right)=\overset{2}{\underset{\alpha =1}{\sum }}n^{\alpha }C_{p}^{\alpha }+T\overset{2}{\underset{\alpha =1}{\sum }}n^{\alpha }g_{xx}^{\alpha }{\left(\frac{dx^{\alpha }}{dT}\right)}_{p}^{2}$$

where *n*^*α*^ is the number of moles in phase α, $\frac{dx^{\alpha }}{dT}$ is the reciprocal slope of the phase boundary and $g_{xx}^{\alpha }$ is the second derivative of the Gibbs energy with respect to composition (always positive). Vertical phase boundaries do not provide any extra contribution to the heat capacity of the two phases, while low values of *dT/dx* strongly contribute to the DSC signal. This readily explains the shape of the DSC signal in Fig. 7. Below the horizontal line the slope is high, and the signal is close to the baseline. After the transition (which is seen as the main unfolding peak) the slope *dT/dx* is high and the signal nearly returns to the baseline, then above 80 °C the slope becomes lower, and the DSC signal exhibits the second peak.

In the amorphous state, the denaturation is a non-equilibrium transition and therefore we do not consider the number of degrees of freedom at these conditions. As it was mentioned above, with decreasing the water content and water activity, the denaturation temperature shifts to higher values. This behaviour illustrates very well the importance of water in the stability of proteins. Since water is needed for denaturation, dehydration makes the protein less susceptible to thermal degradation.

### 3.3 Structural transitions of lysozyme during the thermal treatment investigated by simultaneous SAXS/WAXS

The structure of lysozyme at different levels of hydration during the heat treatment process was investigated using simultaneous SAXS and WAXS. The scattering data cover a q-range from 0.1 nm^-1^ to 30 nm^-1^, in which the intra- and intermolecular correlations of length scale ranging from 0.2 nm to 60 nm could be probed. An overview of the scattering patterns of lysozyme at different water contents at ambient condition has been reported recently [8] which shows that most of the structural information appears in the form of correlation peaks. The peaks correspond to a broad range of characteristic length scales – from those exceeding protein sizes down to typical interatomic distances. For instance, the peaks seen in the SAXS range (q values below 5 nm^-1^) arise from scattering from the overall shape of the protein molecules and from protein-protein correlations. The peaks in the WAXS range (q values above 5 nm^-1^) arise from the intramolecular structure of the protein, including secondary structure and interatomic correlations. In this section, the effect of temperature on the unfolding process of lysozyme will be discussed in detailed for three different hydration regimes: in the liquid state, in the 2-phase system and in the amorphous solid state.

#### 3.3.1 SAXS/WAXS patterns in the liquid state

**Fig. 9:**
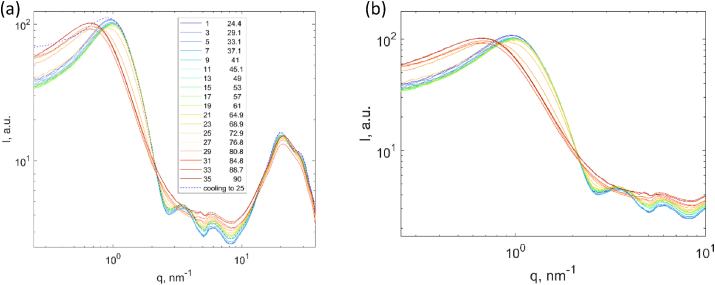
(a) SAXS/WAXS patterns of lysozyme xH_2_O = 85 %wt during heating (solid lines) and after cooling to 25 °C (dash line), (b) Zoom-in SAXS patterns of the same data set for a clear observation.

An example of the scattering patterns of lysozyme at 80 wt % of water as a function of the temperature is shown in Fig. 9. For lysozyme at ambient temperature, in the SAXS range, these patterns consist of a broad peak at q = 1 nm^-1^ which is assigned as the protein-protein interaction, followed by a peak at q = 3.5 nm^-1^ which is a first sub-maxima of the form factor of native lysozyme in solution. In the WAXS regime, by applying the deconvolution procedure reported in our previous work [8], different peak positions related to internal structure of lysozyme molecules could be identified such as: q = 5.5–5.8 nm^-1^ (second peak of the form factor including the hydration shell), q = 6.0–6.2 nm^-1^ (interactions between a helixes), q = 7 - 12 nm^-1^ (secondary structures), q = 14-15 nm^-1^ (hydrophobic groups) and q = 17–19 nm^-1^ (water and hydrogen bonded atoms).

In order to have a better observation of these features, a zoom-in of the scattering patterns for SAXS range is added. In the SAXS range, the peak at q = 1 nm^-1^ shifts to higher q-values with increasing the temperature from 25 °C to 45 °C while no temperature dependence of peak positions is observed at higher q-values. From 45 °C–67 °C, no clear change was observed in the SAXS range. Above 67 °C where the temperature approaching the unfolding of lysozyme, the peak at q = 3.5 nm^-1^ starts to disappear which demonstrates that the protein molecules start to lose their native conformations. In parallel, a shift to lower q-values for the peak at q = 1 nm^-1^ was observed together with an increase in the intensity at the q = 0.1 nm^-1^. It might be because of the change of monomer shape from a compact structure to a more opened structure upon heating as well as formation of the aggregates. As the sample undergoes cooling back to ambient temperature, most of the features were completely recovered to its original state. This demonstrates that the unfolding is reversible. It should also be noticed that the intensity at q = 0.1 nm^-1^ does not decrease which indicates that a small fraction of the aggregates was formed irreversibly.

The reversible thermal unfolding of lysozyme has been reported previously by Hiral et al. [26] using simultaneous SAXS and WAXS. They have demonstrated that the radius of gyration (*R*_*g*_) of protein increases during heating, which was caused by the transition of small compact ternary structure to an opened conformation due to collapsed ternary structure. The change of *R*_*g*_ was found to be strongly coupled with the values obtained from DSC measurements. In addition, these opened conformations facilitate the exposure of the hydrophobic area of the protein surface and strongly affect the heat capacity change of the unfolding process [13].

#### 3.3.2 SAXS patterns in the amorphous solid state

In our previous study we suggested that the protein molecules in the solid amorphous state at the ambient condition can have different short-range 3D arrangement such as HCP or FCC or random close packing which can vary between them depending on the water content [8]. The water content threshold at which the structural transition occurs is around 11 wt% which also coincides with the onset of the glass transition of lysozyme [23,27]. The results from SAXS/WAXS data strongly support our hypothesis that protein molecules in the solid state adopt structures that can continuously fill the space to remove the protein-air interface that may be formed upon dehydration. Here we will present the SWAXS data on thermal denaturation of lysozyme in the water regimes below and above 11 wt%.

**Fig. 10:**
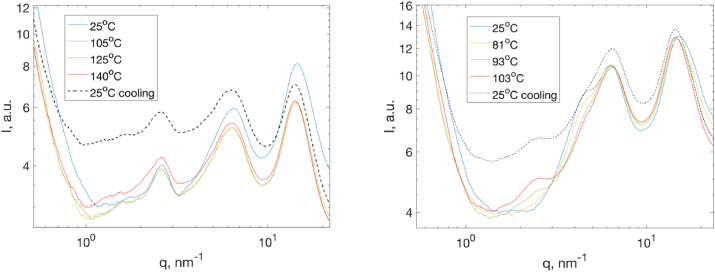
Temperature dependence of scattered intensity I(q) of lysozyme for (a) 2.5 wt% and (b) 16.5 wt% of water content. The dashed line represents the scattering pattern of lysozyme after cooling to 25 °C.

At water content 2.5 wt% where the sample is in the solid amorphous state, the scattering curves show a power law dependence of the intensity at q < 1 nm^-1^ with a slope of −4 (Fig. 10a). The presence of the so-called Porod slope is caused by the scattering from the solid protein - air interface of lysozyme powder particles. At q > 1 nm^-1^ various correlation peaks can be identified and was reported in our previous work for ambient temperature: at q = 2.5 nm^-1^ which corresponds to a distance of d = 2.5 nm arising from protein – protein interaction in the solid state, q = 6.1 nm^-1^ (d = 1 nm) corresponding to interactions between α helixes, q = 14 nm^-1^ (d = 0.45 nm) and q = 18 nm^-1^ assigning to interatomic distances related to hydrophobic groups and water/hydrogen bonded atoms, respectively. During heating as well as after cooling to ambient condition, no pronounced structural changes were observed from the SAXS/WAXS data. It should be noticed that, upon strong dehydration, the secondary structure of protein molecules undergoes dramatic change as reported from other techniques Raman and FTIR [5,6]. At these conditions, the protein molecules are tightly packed to decrease the protein-air interface and therefore the transition to a denatured state might not necessarily be accompanied with such a dramatic change in the molecule shape as in dilute solution.

Fig. 10 b represents the scattering patterns of lysozyme at the water content of 16.5 wt% as a function of the temperature. At low q values, the intensity has the same power law dependence on q as at lower water contents. However, at higher q values, the peak at q = 2.5 nm^-1^ is absent and a new peak at q = 4.5 nm^-1^ appears in the temperature range below 80 °C. As the temperature increases above 80 °C, the peak intensity at q = 4.5 nm^-1^ starts to drop while a new peak at q = 2.5 nm^-1^ starts to develop. These structural patterns, which are similar to those having the water content below 11 wt%, are preserved after cooling to 25 °C which means that the unfolding process is irreversible as revealed by calorimetric measurements.

It is interesting to observe that the scattering patterns obtained by dehydrating lysozyme to the dried state at ambient condition have similarities to those obtained by heating the amorphous solid samples at higher water contents. The distortion of the overall structure caused by dehydration at ambient condition led to reversible changes of the protein molecules structures. However, the changes observed upon increase of temperature in hydrated solid state seem to be irreversible.

#### 3.3.3 SAXS patterns in the two-phase regime

**Fig. 11:**
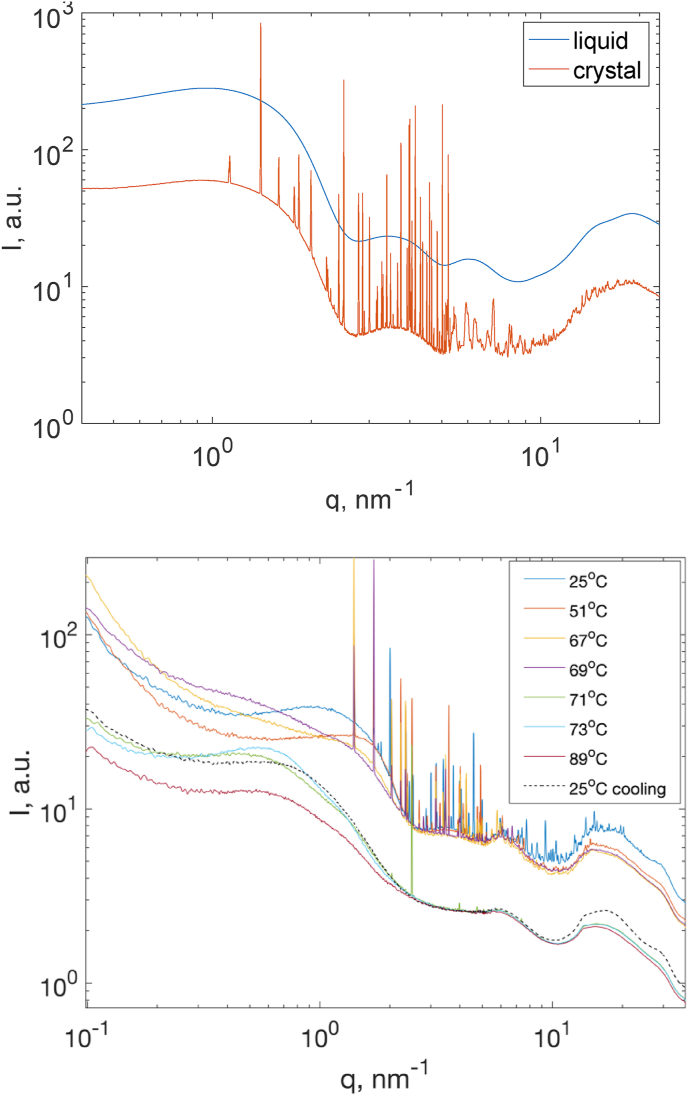
(a) SAXS patterns of lysozyme at the water content of 53 wt% at 25 °C. The blue and red lines represent the data from the top and bottom phase, respectively. (b) Temperature dependence of scattered intensity *I(q)* of lysozyme at the water content of 53 wt% in the crystalline phase. The dashed line represents the scattering pattern of lysozyme after cooling to 25 °C. The scattering curves with Bragg peaks are grouped together for an easier observation. (For interpretation of the references to colour in this figure legend, the reader is referred to the Web version of this article.)

In the intermediate water regime between 40 wt% and 55 wt%, lysozyme undergoes a phase separation. Fig. 11a represents the scattering patterns from two phases of lysozyme from a sample with 53 wt% of water. In the top phase, typical scattering patterns of protein in highly concentrated solution could be observed. The peak at 3.5 nm^-1^ originates from the first maximum of the monomer form factor of lysozyme in the native state and is also found in the scattering pattern from the dilute lysozyme solution, as well as from the PDB structure of lysozyme. In the bottom phase, numerous Bragg peaks were present in a broad q-range from q = 1 nm^-1^ in the SAXS range up to the WAXS range which are due to the lysozyme being partly crystallized. The “baseline” under the Bragg peaks is identical from the data obtained at the liquid phase.

As the temperature increases, various changes in the scattering patterns could be observed (Fig. 11b). When heating up to 60 °C, the only change in the structure patterns is that the peak at q = 1 nm ^-1^ shifts to higher q values. At above 60 °C, the scattered intensity at low q increases and the peak around q = 1 nm ^-1^ becomes broader. In parallel, the number of the Bragg peaks and their intensity decreases significantly. Moreover, the intensity of the peak at q = 3.5 nm ^-1^ starts to decrease. At around 70 °C, all the Bragg peaks as well as the peak at q = 3.5 nm ^-1^ disappeared while the peak at q = 1 nm ^-1^ shifts to lower q values. As the temperature increases above 70 °C the intensity of the peak at q = 1 nm ^-1^ decreases while at lower q values an increase of the intensity is observed. After cooling the sample to ambient temperature, the scattering pattern does not recover to their initial state, only the peak at q = 1 nm^-1^ partly recovered to a level which is similar to the pattern at 70 °C.

When the temperature is below 60 °C, which is still smaller than the unfolding temperature as well as the temperature of melting of crystal, it is clear that the native state of lysozyme as well as the structure of crystal are intact. The only change in the scattering patterns occurs around q = 1 nm^-1^ in which a small shift to higher q values is observed with increasing the temperature, which was also observed in the liquid state.

Above 60 °C two processes happen in parallel: unfolding of the protein, which results in a decrease of the peak intensity at q = 3.5 nm^-1^ which represents the 1st sub maxima of the form factor of native lysozyme, and melting of the crystals as the number and the intensity of Bragg peaks decreases.

It is clear that the unfolding of the protein in the crystal state is irreversible. It could be explained by the fact that in this concentration range the crowding can promote irreversible formation of aggregates in the denatured protein solution.

### 3.4 Raman spectroscopy

#### 3.4.1 Analysis of the amid I band region (1500–1700 cm^-1^)

The amid-I band corresponds mainly to the C=O stretching vibration of amide groups with a small contribution from the N−H in-phase bending and C−N out-of-phase stretching and C−C−N deformation [28,29]. The contribution related to C=O vibrations is highly sensitive to the secondary structure stabilized by C=O…H hydrogen bonding. Weakening of the hydrogen bonding interactions results in a shift of Amid-I frequencies to higher values. The localization of this amide I band around 1650–1660 cm^−1^ is considered as the signature of the native α-helix secondary structure [30]. The band observed around 1670–1675 cm^−1^ was assigned to the intermolecular β-sheet interactions, and then was considered as a probe to detect molecular aggregation through the increase of its intensity [30,31]. Other secondary structural features in higher spectral regime of 1680–1695 cm^−1^ are from weakly hydrogen bonded or nonforming interstrand hydrogen bonded amide groups corresponding to loose β strands, intermolecular β sheet structures, polypiroline II and disordered structures.

A data treatment which consists of a normalization, follows by a subtraction has been performed to investigate the temperature dependence of the 1500–1700 cm^-1^ spectral region.

- Total normalization: the intensities are first normalized to the intensity at 1003 cm^-1^ which corresponds to the vibration of Phe ring.
- Local background subtraction: the total normalized intensities were then subtracted to the intensity at the minimum intensity 1590 cm^-1^

**Fig. 12:**
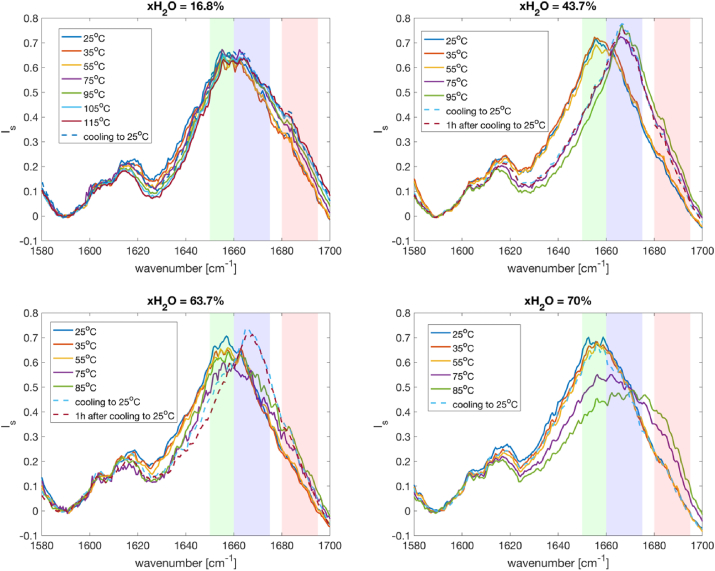
Raman spectra for lysozyme in the Amide – I spectra region at different water contents indicated in the figure.

Fig. 12 represents the temperature dependence of Raman spectra in the Amide I spectral region for different water contents indicated in the figure. In the solid amorphous sample (xH_2_O = 16.8 %wt), the difference in the peak intensities and peak positions during the heating upon the denaturation temperature and after cooling to ambient temperature is not pronounced. It has been considered that in this low water regime, the protein molecules are in a distorted conformation in which the amount of β sheet structure is dominated [6,8]. Therefore, no significant addition of β sheet caused by temperature could be observed.

In the water content of 43.7 wt% in which two phases coexist, a clear shift of the amid I band from 1655 cm to 1 to 1667 cm^-1^ is observed during the heating process. The spectra obtained after cooling to ambient temperature is found at the same position as the spectra at high temperature. The shift of frequency from α helixes to β sheet structures indicates that the protein molecules undergo unfolding which is an irreversible process as the spectra do not recover to the original state. It implies that the denaturation process in irreversible.

The sample in the water content of 63.7 wt% which is laying on the border of the one-phase and two-phases systems shows similar behavior in the Raman spectra as to the data obtained on the two-phase system. In particular, the peak intensity at 1650 cm^-1^ decreases with increasing temperature together with a peak broadening toward higher frequency. The drop in intensity at the 1650 cm^-1^ and the shift of the peak to higher frequencies indicate the transformation of part of the secondary structure of lysozyme from α-helix to β-sheet. This transformation is governed by a kinetics behaviour as it apparently continues upon cooling. After cooling to 25 °C, the 1650 peak shifts to the 1667 cm^-1^ position indicating that the process is irreversible.

Raman spectra of liquid samples at higher water contents show similar unfolding behaviors during heating compared to the 63.7 wt% water content sample such as drop in intensity at the peak position 1650 cm^-1^ and broadening of the peak toward higher frequency. However, after cooling to ambient temperature for higher water content samples, the peak position shifts back to the initial position, which indicates that the unfolding is reversible.

The difference in the reversibility of unfolding behaviour of lysozyme in the regime of 63.7%–70% could be interpreted by the fact that lysozyme can be misfolded which promote the formation of irreversible aggregates. In addition, the crowded condition in these samples might play a role in facilitating the formation of aggregates.

#### 3.4.2 Analysis of the N−Cα−C stretching vibration

Another feature that was used to study the thermal unfolding process of lysozyme in different hydration levels is the N−Cα−C stretching vibration mode. This mode is located in the spectral regime of 935 cm^-1^ and is sensitive to the presence of α-helix secondary structures in the Raman spectrum [[32], [33], [34], [35]].

**Fig. 13:**
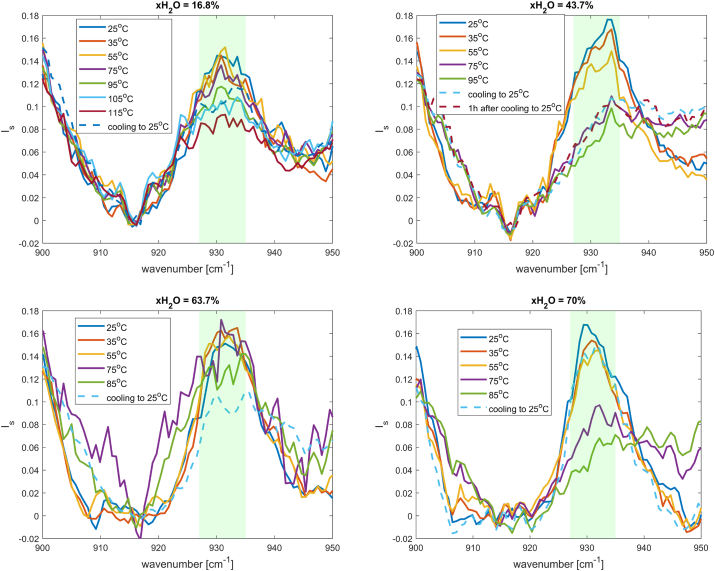
Raman spectra of lysozyme in the α-helix marker mode ν(N−Cα−C) spectral region at different water contents indicated in the figure.

In Fig. 13, subtracted intensity at 915 cm^-1^ Raman spectra of lysozyme in the α-helix marker mode ν(N−Cα−C) spectral region at different water contents were plotted. As the protein molecules undergo unfolding upon heating, the amount of the α-helixes decreases, which results in a decrease of the intensity of the peak at 935 cm^-1^. Upon cooling to ambient temperature, the protein does not recover back to its native state in the water regime below 65 %wt. Above this threshold, a partial or full recovery of the native state could be observed.

Raman studies in the spectral regimes strongly support our hypothesis of the water dependence of the mechanism of the thermal unfolding process of lysozyme as revealed by the DSC measurements.

## 4 Conclusions

We have investigated the reversibility of the thermal denaturation of lysozyme for different hydration levels using a combination of experimental techniques such as DSC, SAXS/WAXS and Raman spectroscopy and revealed strong water content dependence of the thermal denaturation mechanisms:

- In the solid amorphous state (xH_2_O < 37 wt%) the unfolding process is irreversible and can be described by the kinetic approach. In these conditions, the low value of the water activity leads to a substantial increase of the denaturation temperature of lysozyme. A slower dynamics of the system in combination with a deficit of water needed to unfold the protein improves the stability of the protein against thermal denaturation. The analysis of the activation energies shows existence of two processes suggesting either two different populations of proteins or two different denaturation mechanisms.
- In the liquid state (xH_2_O > 60 wt%) the process is reversible, and the thermodynamic equilibrium approach is used to describe the process.
- In the intermediate range of water between 37 wt% and 60 wt%, the system is phase separated and denaturation involves two processes: melting of protein crystal and unfolding of individual molecules.

The experimental data were used to establish a detailed lysozyme – water phase diagram, which gave a detailed picture of the hydration effect on the thermal stability of the protein. Such understanding can have an important impact on pharmaceutical and biotechnological applications such as preservation of biomaterials and optimization of solid-state pharmaceutics where the materials are exposed to different temperature and moisture conditions.

## Declaration of competing interest

We declare no conflict of interest.
